# Hippocampal dosimetry correlates with the change in neurocognitive function after hippocampal sparing during whole brain radiotherapy: a prospective study

**DOI:** 10.1186/s13014-015-0562-x

**Published:** 2015-12-10

**Authors:** Ping-Fang Tsai, Chi-Cheng Yang, Chi-Cheng Chuang, Ting-Yi Huang, Yi-Ming Wu, Ping-Ching Pai, Chen-Kan Tseng, Tung-Ho Wu, Yi-Liang Shen, Shinn-Yn Lin

**Affiliations:** Department of Radiation Oncology, Chang Gung Memorial Hospital and Chang Gung University, #15, Wenhua 1st Rd., Kwei-Shan Hsiang, Taoyuan, Taiwan; Department of Medical Imaging and Radiological Sciences, College of Medicine, Chang Gung University, Taoyuan, Taiwan; Department of Occupational Therapy, Division of Clinical Psychology, Master of Behavioral Sciences, College of Medicine, Chang Gung University, No. 259, Wen-Hwa 1st Road, Kwei-Shan Tao-Yuan, 333 Taiwan; Department of Neurosurgery, Chang Gung Memorial Hospital and Chang Gung University, #5, Fu-Shin Street, Kwei-Shan Hsiang, Taoyuan, Taiwan; Department of Medical Imaging and Intervention, Chang Gung Memorial Hospital and Chang Gung University, #5, Fu-Shin Street, Kwei-Shan Hsiang, Taoyuan, Taiwan; Graduate Institute of Epidemiology and Preventive Medicine, College of Public Health, National Taiwan University, Taipei, Taiwan

**Keywords:** Brain metastasis, Whole brain radiotherapy, Neurocognitive functions, Hippocampus, Hippocampus sparing during whole brain radiotherapy, Hippocampal dosimetry

## Abstract

**Background:**

Whole brain radiotherapy (WBRT) has been the treatment of choice for patients with brain metastases. However, change/decline of neurocognitive functions (NCFs) resulting from impaired hippocampal neurogenesis might occur after WBRT. It is reported that conformal hippocampal sparing would provide the preservation of NCFs. Our study aims to investigate the hippocampal dosimetry and to demonstrate the correlation between hippocampal dosimetry and neurocognitive outcomes in patients receiving hippocampal sparing during WBRT (HS-WBRT).

**Methods:**

Forty prospectively recruited cancer patients underwent HS-WBRT for therapeutic or prophylactic purposes. Before receiving HS-WBRT, all participants received a battery of baseline neurocognitive assessment, including memory, executive functions and psychomotor speed. The follow-up neurocognitive assessment at 4 months after HS-WBRT was also performed. For the delivery of HS-WBRT, Volumetric Modulated Arc Therapy (VMAT) with two full arcs and two non-coplanar partial arcs was employed. For each treatment planning, dose volume histograms were generated for left hippocampus, right hippocampus, and the composite hippocampal structure respectively. Biologically equivalent doses in 2-Gy fractions (EQD_2_) assuming an alpha/beta ratio of 2 Gy were computed. To perform analyses addressing the correlation between hippocampal dosimetry and the change in scores of NCFs, pre- and post-HS-WBRT neurocognitive assessments were available in 24 patients in this study.

**Results:**

Scores of NCFs were quite stable before and after HS-WBRT in terms of hippocampus-dependent memory. Regarding verbal memory, the corresponding EQD_2_ values of 0, 10, 50, 80 % irradiating the composite hippocampal structure with <12.60 Gy, <8.81, <7.45 Gy and <5.83 Gy respectively were significantly associated with neurocognitive preservation indicated by the immediate recall of Word List Test of Wechsler Memory Scale-III. According to logistic regression analyses, it was noted that dosimetric parameters specific to left sided hippocampus exerted an influence on immediate recall of verbal memory (adjusted odds ratio, 4.08; *p*-value, 0.042, predicting patients’ neurocognitive decline after receiving HS-WBRT).

**Conclusions:**

Functional preservation by hippocampal sparing during WBRT is indeed achieved in our study. Providing that modern VMAT techniques can reduce the dose irradiating bilateral hippocampi below dosimetric threshold, patients should be recruited in prospective trials of hippocampal sparing during cranial irradiation to accomplish neurocognitive preservation while maintaining intracranial control.

**Trial registration:**

Current Controlled Trials NCT02504788

**Electronic supplementary material:**

The online version of this article (doi:10.1186/s13014-015-0562-x) contains supplementary material, which is available to authorized users.

## Background

Brain metastasis is usually a dismal diagnosis affecting 200,000 Americans each year and up to 30 % of patients with cancer [1]. Traditionally, whole brain radiation (WBRT) with or without surgical resection has ever been the treatment of choice for patients with solitary brain metastasis and WBRT alone for those with multiple brain metastases; WBRT has long been a practical therapeutic modality for various settings of management in radiation oncology [2, 3]. For instance, the indications for WBRT include overt brain metastasis/metastases, the setting of prophylactic cranial irradiation (PCI) used for patients with limited-stage small cell lung cancer, and even some patients with extensive-stage small cell lung cancer [4]. Although control of the metastatic brain lesion(s) by WBRT might be generally the most important factor for stabilizing neurocognitive functions [5], paradoxical decline in neurocognitive functions (NCFs) can also occur as a sequel of WBRT which cannot be negligible. Actually the time course of WBRT-induced NCF decline can vary greatly according to the specific neurobehavioral domains measured. Early neurocognitive decline occurs within the first one to four months after the patient receives the course of WBRT for brain metastases [6]. The domains of radiation-related neurocognitive decline should mainly encompass immediate and delayed verbal memory with or without non-verbal memory [7, 8]. According to the study reported by Sun et al. [7], verbal memory function was most likely to deteriorate significantly after whole brain irradiation; however, general nonspecific cognitive functions and quality of life were not adversely influenced after receiving.

Over the past several decades ago, it has been established that hippocampus plays an essential role in memory function [9]. Additionally, not little evidence suggests that impaired hippocampal neurogenesis resulting from cranial irradiation [10–13] should be strongly associated with NCF impairment [9, 14]. Moreover, a few studies demonstrated that the isodose distribution irradiating the hippocampus is definitely related to NCFs after cranial irradiation in patients with primary brain tumors [15–17] or in those with nasopharyngeal carcinoma [18, 19]. As a result, it has been hypothesized that conformal hippocampal sparing during the course of WBRT (HS-WBRT) would provide meaningful preservation in terms of cognitive function [20–22]. Additionally, thanks to the great advancement in radiotherapy techniques such as volumetric-modulated arc therapy (VMAT) and helical tomotherapy, it is feasible to achieve conformal avoidance of the centrally located hippocampus while maintaining uniform dose delivery to the remaining brain [23–25]. Our pilot study and unpublished preliminary results also have clearly suggested that HS-WBRT should be a feasible and recommended technique which provides satisfactory intracranial control and preserves responsible NCFs simultaneously in patients with favorable prognosis harboring a limited burden of brain metastases. Besides, we should pay attention to the concern whether hippocampal sparing would increase the potential risk of metastases occurring inside or close to the hippocampus. According to the study reported by Ghia et al. [20], a 5-mm margin around the hippocampus for conformal avoidance during WBRT represents an acceptable risk. Furthermore, the sites of relapse after HS-WBRT uncommonly develop perihippocampally if patients undergoing HS-WBRT indeed harbor a limited burden of brain metastases.

Therefore, the current study not only aims to confirm the impact of the delivery of HS-WBRT on the extent of NCF changes in patients receiving hippocampal sparing during the WBRT course using VMAT technique, but also to investigate especially the relationship between hippocampal dosimetry and neurocognitive outcomes,, as measured with objective neurocognitive assessment tools.

## Methods

### Patient selection

Patients with primary lung cancer referred for PCI or adults with pathologically-confirmed non-hematopoietic malignancy and brain metastasis who had fair to good performance status represented by Karnofsky Performance Status (KPS) score 70 or Eastern Cooperative Oncology Group (ECOG) score 2 were eligible for this study. Additionally, the number and extent of brain metastatic lesions should be no more than three metastatic foci with a limitation of greatest diameter less than 4 cm. In addition, this inclusion criterion was confirmed in patients by performing brain magnetic resonance imaging (MRI) after they underwent craniotomy with tumor removal and before the course of WBRT if potentially eligible patients were surgically resected cases. Thus, in addition to patients referred for PCI, most of our enrolled patients should correspond with the definition of oligometastatic brain disease, implying that the number of metastatic foci was three or less shown on brain MRI [26, 27]. Accordingly, patients must fall into RTOG recursive partitioning analysis (RPA) class I or II [28, 29]. A table describing the characteristics of our patient cohort is summarized in Additional file 1: Table S1.

Of note, there are other potential patient-, disease-, and treatment factors which also play a role in affecting NCF change, such as the extent of brain edema caused by the metastatic brain focus *per se* or surgical intervention, the confounding effect of increased intracranial pressure (IICP), nutritional condition, and electrolyte imbalance. Nevertheless, our pre-defined strict inclusion criteria have focused our enrolled patients on those with satisfactory performance status and a limited number and burden of metastatic brain foci. As a consequence, it is indeed assumed that the impact of potential confounders can be kept as negligible as possible provided that the enrolled patients really meet our eligibility criteria (unpublished results).

Given the concerns of safety issue whether reducing the dose delivered to hippocampal areas below the therapeutic level might potentially increase the risk of hippocampal metastasis, patients with MRI-identified metastasis within 5 mm perihippocampally were excluded. Other exclusion criteria include clinical suspicion of leptomeningeal spreading, a history of prior radiotherapy including stereotactic radiosurgery delivered to brain/head region for any reasons, and contraindication for receiving contrast-enhanced MRI examination.

The study protocol had been approved by the institutional review board (IRB) at our institute (IRB 101-4151B and 103-1090C) and written informed consent was obtained from each enrolled and eligible patient or the person authorized to give consent. Of note, the current study is a principle investigator (PI)-initiated study; neurosurgeons and radiation oncologists constitute the main investigators taking responsibility for recruiting appropriate patients for this prospective study.

### Neurocognitive assessments

No matter whether the enrolled patients are surgically resected cases or not, Gadolinium-enhanced MRI should be obtained within one month prior to the planned course of HS-WBRT in order to ensure that potentially eligible patients had no any detected brain metastasis within a 5-mm margin around either hippocampus on MRI. Similarly, relevant physical examination and staging oncological surveys should be performed within 30 days before the initiation of HS-WBRT course. Most importantly, all participants must receive the baseline neurocognitive assessment (mentioned below), which was administered within two weeks before the start of HS-WBRT course. Besides, the time interval between brain MRI examination and baseline neurocognitive testing is as short as possible, preferably within two weeks.

A selective neurobehavioral test battery is pre-determined and administered in this prospective study as shown in Table 1. The neurobehavioral measures mainly evaluate several domains assumed to be sensitive to the tumor involvement and the impact of cranial radiation therapy; all tests were administered by a trained clinical research associate under the supervision of a neuropsychologist.

**Table 1:** The neurocognitive test battery administered at baseline and 4 months after the course of HS-WBRT

Neurocognitive Function Test	Domain
Memory	
WMS-III Word Lists (immediate, delayed recall and recognition)	Verbal memory
WMS-III Visual Reproduction (immediate, delayed recall and recognition)	Visual memory
WAIS-III Digit Span	Working memory
Processing speed	
WAIS-III Digit Symbol	Psychomotor speed
WAIS-III Symbol Search Tests	Psychomotor speed
Executive Functions	
Wisconsin Card Sorting Test	Cognitive flexibility

Abbreviations: *WMS* wechsler memory scale, *WAIS* wechsler adult intelligence test

Since the delivery of HS-WBRT instead of conventional WBRT aims to diminish the extent of neurotoxic impact on the hippocampus, which is significantly associated with memory functions [9, 16, 22, 30], three main aspects of NCF including memory functions, executive functions, and psychomotor speed are thus evaluated. First, regarding the domain of memory, the selected subtests of the Wechsler Memory Scale- 3rd edition (WMS-III) were used to evaluate patients’ verbal and non-verbal episodic memory. In terms of verbal memory, the Word Lists (WL) Subset was used, while the Visual Reproduction (VR) Subtest was employed to assess the capability of non-verbal memory. Second, concerning the aspect of executive functions, the Modified Card Sorting Test [31] is used to assess both conceptual formation and mental shifting which have been documented to be the major components of executive functions. Conceptual formation is represented by the score obtained from the number of completed categories (CC) and mental shifting is depicted by the score from the number of preservative errors (PE). Besides, the Digit Span Subtest (DS) subset of the Wechsler Adult Intelligence Scale- 3rd edition (WAIS-III®) [32] was used to examine the verbal working memory. Finally, in order to determine the patients’ performance on the psychomotor speed, Psychomotor Speed Index (PSI), which is derived from the composite score of Digit Symbol Coding Subtest (DSS) and Symbol Searching Subtest (SS) of the WAIS-III®, was used.

### Treatment planning and delivery

**Fig. 1:**
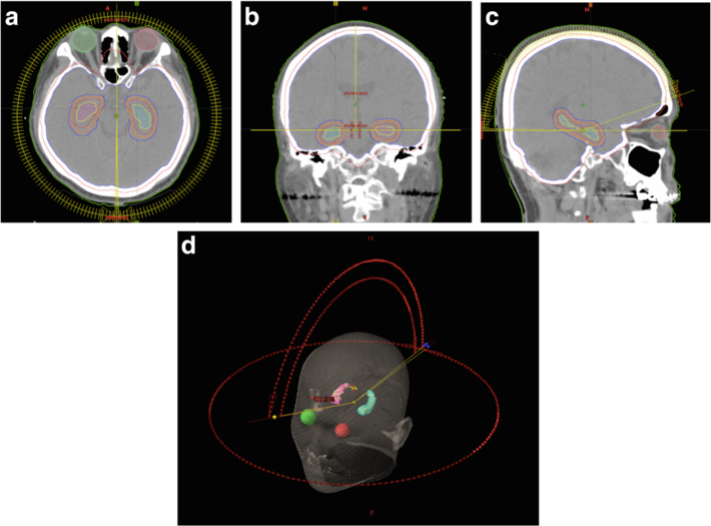
An example of hippocampi contouring was demonstrated in (**a**) Axial, (**b**) Coronal and (**c**) Sagittal views. The bilateral hippocampal structures were contoured in yellow color. The zone for hippocampus avoidance (HA zone) was marked in orange. **d** Treatment planning was designed and arranged by four arcs in a VMAT plan, in which two full arcs and two non-coplanar partial arcs were employed

All enrolled patients underwent a computed tomography (CT) simulation scan encompassing the entire head region with 1.25-mm slice thickness using a thermoplastic mask for immobilization. Bilateral hippocampal areas were contoured on T1-weighted sequence of axial magnetic resonance imaging (MRI) with gadolinium contrast enhancement. Hippocampal contouring was created and confirmed by a same experienced neuroradiologist. Figure 1(a)-(c) displays that appropriate anatomical contouring was also modified and verified by using T1-weighted MRI coronal and sagittal sequences. Furthermore, the hippocampal avoidance zone (HA zone) was generated by expanding the hippocampal outlines with a margin of 5-mm volumetrically to allow for the sharp dose fall-off between bilateral hippocampal structures and the planning target volume (PTV) of whole brain. [23].

To achieve conformal hippocampal sparing during the delivery of WBRT, the technique of volumetric modulated arc therapy (VMAT) was employed with using linear accelerator. Two full arcs and two non-coplanar arcs of VMAT were designed in VMAT treatment planning as shown in Fig. 1(d). The prescribed dose was 25Gy in 10 fractions for prophylactic WBRT or 30 Gy delivered in 10 to 12 fractions for therapeutic or adjuvant WBRT. General principles for target coverage are 100 % coverage for tumor bed and/or gross metastatic tumor, 95 % coverage for clinical target volume (CTV) and 90 % coverage for planning target volume (PTV) with hot spots receiving less than 115 % of the prescription dose.

Furthermore, In order to reflect the corresponding effects of different levels of biologically equivalent doses, all the doses described as follows would be transformed to the equivalent doses in 2-Gy fractions (EQD_2_) assuming α/β = 2 Gy. Regarding the hippocampal dosimetry, the percentage of a hippocampal volume receiving at least m% of the prescribed dose was presented as V_m%_; the dose irradiating at most q% of the hippocampal volume was indicated by D_q%_. For example, D_40%_ corresponds to the EQD_2_ irradiating 40 % of the hippocampal volume of interest. Owning to the principle of hippocampus sparing which must indicate a sharp dose fall-off within the small volume of the hippocampal structure, it should be anticipated that the larger the q% is, the lower the corresponding dose of D_q%_ is.

Generally, the hippocampus sparing in our study would be achieved by limiting V_40%_ less than 15 % or as low as achievable while maintaining acceptable target coverage. The dose volume histograms (DVH) for each patient were generated for left hippocampus, right hippocampus, and the composite structure of bilateral hippocampi, individually. Before performing the analyses addressing the correlations between dosimetric parameters and NCF scores of interest, we retrieved from the Eclipse treatment planning system quite a few dosimetric variables, including the hippocampal volume of interest and the various dose levels irradiating the hippocampal structure. All physical doses were converted to biologically equivalent doses in 2-Gy fractions (EQD_2_) assuming an α/β ratio of 2 Gy.

### Statistical analysis

The primary neurocognitive endpoint was delayed recall, as determined by the change/decline in either verbal memory [WMS III – Word Lists (WL)] or non-verbal memory [WMS III - Visual Reproduction (VR)] from baseline pre-WBRT assessment to 4 months after the start of HS-WBRT. As a result, to compare patients’ NCFs before and after the HS-WBRT course, the repeated measure analysis of variance (ANOVA) was utilized. The statistical significance was set as *p* value of less than 0.05 after the Bonferroni adjustment.

Concerning neurocognitive outcomes, each patient served as his/her own control, therefore the change in NCF scores for each subtest from baseline to 4-month after the HS-WBRT course was calculated as the post-WBRT normalized score minus the baseline normalized one. By comparing the NCF scores between pre-RT and post-RT regarding the HS-WBRT course, patients in this study were dichotomized into binary groups according to the median value of each hippocampal dosimetric parameter. If the change in the NCF score under investigation in one patient shows neurocognitive improvement, this patient will be categorized as the group with NCF preservation group; all the others will be among the group without experiencing NCF preservation

Before performing the analyses addressing the correlations between dosimetric parameters and NCF scores of interest, the median and mean volumes for individual or composite hippocampal structures were extracted from Eclipse treatment planning system and subsequently converted into EDQ_2_ values as stated previously. First, according to the medians of the above various levels of EQD_2_ values, within each dosimetric parameter, the dosimetric data were categorized into a higher dose subgroup and a lower one, using the median as the cut-off point. Second, the changes in NCF scores between baseline and 4-months after the HS-WBRT course were classified as a subgroup of patients experiencing neurocognitive preservation and the other patient subgroup without achieving NCF preservation. Neurocognitive preservation was defined that the change in the specific NCF score of interest was more than zero. Finally, the association between each dosimetric parameter of interest and various NCF scores were extensively investigated by using the chi-square test. Binary logistic regression was also employed to investigate the independent effects of hippocampal dosimetric parameters on NCF change (decline vs. no decline); adjusted ORs were computed to stand for the independent effects of the dosimetric parameters of interest after controlling for the patient’s age at study enrollment and whether craniotomy with tumor removal was performed or not before undergoing the course of HS-WBRT.

## Results

### Neurocognitive outcomes directly related to hippocampus-dependent memory functions

All recruited patients in this prospective study should receive baseline NCF assessment before undergoing the subsequent HS-WBRT course. Overall compliance with NCF testing was 68 % at months after the initiation of HS-WBRT course. The majority of failure to achieve compliance should be ascribed to patient-related factors such as an unsatisfactory performance status impeding the administration of neurocognitive assessment. Consequently, there were totally 24 patients whose post-treatment follow-up NCF assessment was available. With regard to neurocognitive outcomes in our study, generally NCF scores were relatively stable before and after HS-WBRT, in terms of immediate and delayed verbal/non-verbal memory. Remarkably, neurocognitive stabilization was the mainly observed finding rather than NCF decline. For instance, With regard to the verbal memory reflecting the episodic memory function of left hippocampus, no significant difference in the scaled scores of the short-term or long-term memory on the Word List learning (WLL) was observed between before and after the course of HS-WBRT (*F* =1.402, *p* =0.248, for short-term memory; *F* =0.006, *p* =0.969, for long-term memory). Importantly, the change in verbal memory indicated by WLL tends to stabilize and even improve rather than decline. As well, with respect to the non-verbal memory indicating the episodic memory function of right hippocampus, the change in any scaled scores on Visual Reproduction (VR) between pre- and post-WBRT course also tended to become stabilized with somewhat improvement (*F* =1.125, *p* =0.3, for short-term memory; *F* =0.397, *p* =0.535, for long-term memory; *F* =10.18, *p* =0.004, for recognition).

### Performance of VMAT plan and DVH results of the hippocampal structure

**Fig. 2:**
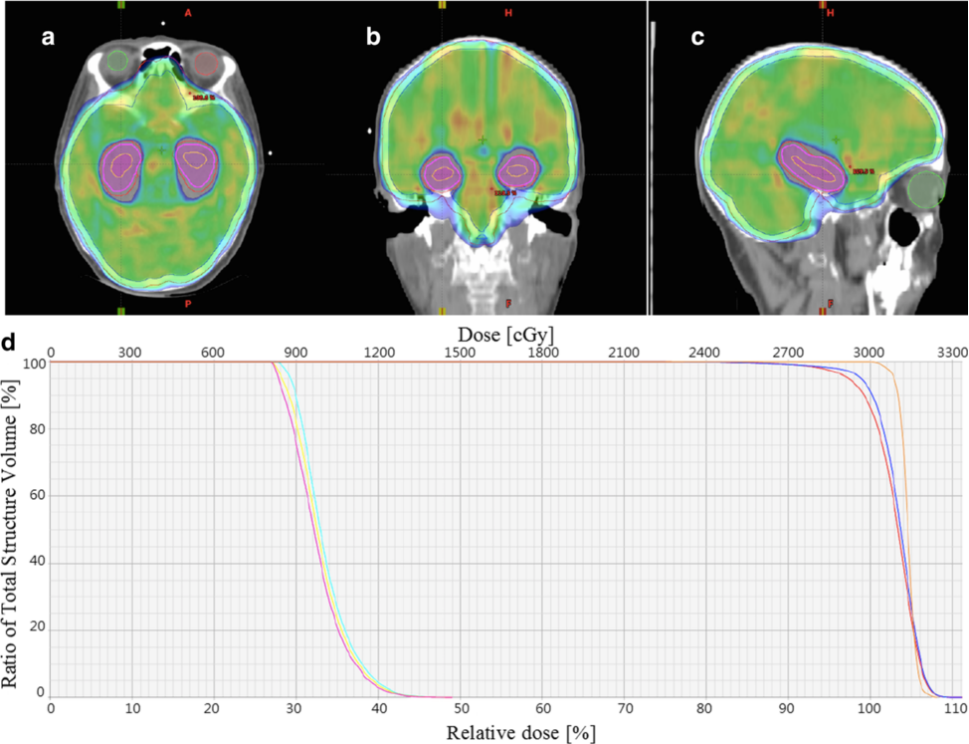
An example of 90 % isodose distribution in the display modes of color wash and dose volume histogram (DVH). **a** Axial, **b** Coronal, **c** agittal views and (**d**) The displayed DVH is in accordance with the prescription of 3000 cGy in physical dose. The yellow curve outlines where the bilateral hippocampal structures are, red for PTV, blue for CTV, and orange for the region of gross tumor or tumor bed

Figure 2 shows that hippocampus sparing was generally accomplished with satisfactory target coverage and conformity in our study. Based on the DVH results in this study, the V_90%_ of PTV ranges from 96.51 to 99.88 % (98.08 ± 0.907 %) and the dose percentages corresponding to the 1c.c. hot spot areas were between 108.64 % and 114.38 % (112.036 ± 1.615).

**Fig. 3:**
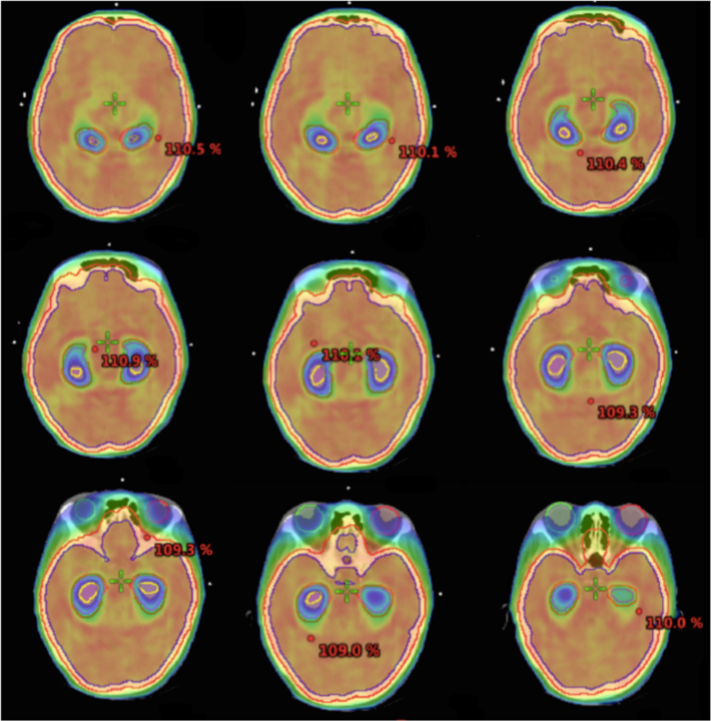
The isodose region without added color display represents the site of hippocampal sparing. The 40 % isodose displayed in color wash indicates where our VMAT treatment plan has attempted to achieve so-called hippocampal sparing

In terms of hippocampus sparing via VMAT achieved in our study, the volume percentage of V_40%_ ranges from 0.24 to 71.44 % (15.42 ± 17.34 %) for left hippocampus and between 0.215 and 63.05 % (15.3 ± 17.528 %) for right hippocampus (Unpublished results). Axial views illustrating different sections of conformal hippocampus avoidance were shown in Fig 3. It apparently demonstrates that normal tissue sparing via our VMAT planning is achieved not only for the hippocampal structure but also for the eyes, while target coverage and conformity are maintained simultaneously.

The descriptive data of hippocampal dosimetric parameters converted to the corresponding EQD_2_ values are listed in Table 2. The volumes of each individual hippocampus range from 2.20 c.c. to 2.79 c.c., with a mean volume of 2.41 c.c. for left hippocampus and 2.71 c.c. for right hippocampus. According to the EQD_2_ dose values of D_20%_, D_40%_, D_50%_ and D_80%_ irradiating the hippocampal structure of interest, it is noted that both left and right hippocampal structures can be spared approximately the similar dose levels in this study, generally below the EQD_2_ value of 8.5 Gy.As for the median EQD_2_ values of maximal and minimal dose levels irradiating the hippocampal structure of interest, they are 12.6 Gy and 5.8 Gy for left hippocampus, and 12.4 Gy and 5.7 Gy for right hippocampus respectively.

**Table 2:** A summary of hippocampal dosimetric parameters used in this study

	Bilateral hippocampi		Left hippocampus		Right hippocampus	
	Mean (95 % CI)	Median	Mean (95 % CI)	Median	Mean (95 % CI)	Median
Volume (c.c.)	4.99 (4.59–5.40)	5.18	2.41 (2.20–2.62)	2.41	2.58 (2.37–2.79)	2.71
Maximum EQD_2_ (Gy)	13.51 (11.85–15.17)	12.64	13.20 (11.50–14.9)	12.41	12.93 (11.64–14.22)	12.64
D_20%_ EQD_2_ (Gy)	8.30 (7.81–8.78)	8.26	8.34 (7.86–8.82)	8.22	8.27 (7.78–8.76)	8.28
D_40%_ EQD_2_ (Gy)	7.65 (7.19–8.11)	7.68	7.67 (7.22–8.12)	7.70	7.63 (7.16–8.10)	7.70
D_50%_ EQD_2_ (Gy)	7.40 (6.96–7.85)	7.45	7.41 (6.97–7.85)	7.48	7.37 (6.92–7.82)	7.47
D_80%_ EQD_2_ (Gy)	6.70 (6.32–7.07)	6.80	6.72 (6.35–7.09)	6.73	6.69 (6.30–7.07)	6.84
Minimum EQD_2_ (Gy)	5.78 (5.51–6.04)	5.82	5.61 (5.04–6.18)	5.73	5.86 (5.58–6.14)	5.90

Abbreviations: *D*_*10%*_ biologically equivalent dose in 2-Gy fractions (EQD_2_) assuming α/β = 2Gy to 10 % of the hippocampus volume of interest (left, right, or bilateral); therefore, D_m%_ is defined as the EQD_2_ to m% of the hippocampal volume of interest; D_100%_ = the EQD_2_ to 100 % of the structure volume, corresponding to the minimal dose received by the hippocampus of interest in our study; *Maximum* the maximal EQD_2_ irradiating the hippocampus volume of interest

### Correlation of Hippocampal dosimetry with neurocognitive functions

First of all, based on the medians of different levels of EQD_2_ values of each dosimetric parameter, the dosimetric data were categorized into two subgroups. Second, as shown in Tables 3 and 4, the changes in neurocognitive scores between baseline and 4-month after the HS-WBRT course were classified as two subgroups according to whether neurocognitive preservation was achieved or not. Finally, the correlations of each dosimetric parameter of interest with various NCF scores were extensively examined and reported.

**Table 3:** Correlation of hippocampal dosimetry with the status of NCF change in verbal memory after HS-WBRT

Dosimetric parameters	Dosimetric cut-off points	NCF Preservation N (%)	No preservation	*p* value
Bilateral hippocampi as a composite structure				
Maximum	≤12.6 Gy	10(83.3 %)	2(16.7 %)	0.004*
	>12.6 Gy	3(25.0 %)	9(75.0 %)	
D_10%_	≤8.81 Gy	9(75.0 %)	3(25.0 %)	0.041*
	>8.81 Gy	4(33.3 %)	8(66.7 %)	
D_20%_	≤8.26 Gy	8(66.7 %)	4(33.3 %)	0.219
	>8.26 Gy	5(41.7 %)	7(58.3 %)	
D_30%_	≤7.95 Gy	8(66.7 %)	4(33.3 %)	0.219
	>7.95 Gy	5(41.7 %)	7(58.3 %)	
D_40%_	≤7.68 Gy	8(66.7 %)	4(33.3 %)	0.219
	>7.68 Gy	5(41.7 %)	7(58.3 %)	
D_50%_	≤7.45 Gy	9(75.0 %)	3(25.0 %)	0.041*
	>7.45 Gy	4(33.3 %)	8(66.7 %)	
D_80%_	≤6.80 Gy	9(75.0 %)	3(25.0 %)	0.041*
	>6.80 Gy	4(33.3 %)	8(66.7 %)	
D_100%_	≤5.83 Gy	9(75.0 %)	3(25.0 %)	0.041*
	>5.83 Gy	4(33.3 %)	8(66.7 %)	
Left hippocampus				
Maximum	≤12.41 Gy	10(83.3 %)	2(16.7 %)	0.004*
	>12.41 Gy	3(25.0 %)	9(75.0 %)	
D_10%_	≤8.75 Gy	9(75.0 %)	3(25.0 %)	0.041*
	>8.75 Gy	4(33.3 %)	8(66.7 %)	
D_20%_	≤8.22 Gy	8(66.7 %)	4(33.3 %)	0.219
	>8.22 Gy	5(41.7 %)	7(58.3 %)	
D_30%_	≤7.94 Gy	8(66.7 %)	4(33.3 %)	0.219
	>7.94 Gy	5(41.7 %)	7(58.3 %)	
D_40%_	≤7.70 Gy	9(75.0 %)	3(25.0 %)	0.041*
	>7.70 Gy	4(33.3 %)	8(66.7 %)	
D_50%_	≤7.48 Gy	9(75.0 %)	3(25.0 %)	0.041*
	>7.48 Gy	4(33.3 %)	8(66.7 %)	
D_80%_	≤6.73 Gy	9(75.0 %)	3(25.0 %)	0.041*
	>6.73 Gy	4(33.3 %)	8(66.7 %)	
D_100%_	≤5.73 Gy	8(66.7 %)	4(33.3 %)	0.219
	>5.73 Gy	5(41.7 %)	7(58.3 %)	

The status of neurocognitive change shown here is according to patients’ performance on the immediate recall of Wechsler Memory Scale-III Word Lists. The association between hippocampal dosimetry and the status of NCF change (preservation or not) in verbal memory 4 months after the HS-WBRT course was evaluated by using chi-square test for the 24 patients in whom post-treatment NCF assessment was available

The N (%) is listed to stand for the number of patients and its corresponding percentage

* indicates that statistical significance is noted

**Table 4:** Association between hippocampal dosimetry and the status of NCF change in Wisconsin Card Sorting Test

Dosimetry	Dosimetric cut-off points	NCF Preservation N (%)	No preservation	*p* value
Bilateral hippocampi as a composite structure				
Maximum	≤12.6 Gy	9(75.0 %)	3(25.0 %)	0.098
	>12.6 Gy	5(41.7 %)	7(58.3 %)	
D_10%_	≤8.81 Gy	7(58.3 %)	5(41.7 %)	1
	>8.81 Gy	7(58.3 %)	5(41.7 %)	
D_20%_	≤8.26 Gy	7(58.3 %)	5(41.7 %)	1
	>8.26 Gy	7(58.3 %)	5(41.7 %)	
D_30%_	≤7.95 Gy	6(50.0 %)	6(50.0 %)	0.408
	>7.95 Gy	8(66.7 %)	4(33.3 %)	
D_40%_	≤7.68 Gy	6(50.0)%	6(50.0 %)	0.408
	>7.68 Gy	8(66.7 %)	4(33.3 %)	
D_50%_	≤7.45 Gy	7(58.3 %)	5(41.7 %)	1
	>7.45 Gy	7(58.3 %)	5(41.7 %)	
D_80%_	≤6.80 Gy	7(58.3 %)	5(41.7 %)	1
	>6.80 Gy	7(58.3 %)	5(41.7 %)	
D_100%_	≤5.83 Gy	7(58.3 %)	5(41.7 %)	1
	>5.83 Gy	7(58.3 %)	5(41.7 %)	
Left hippocampus				
Maximum	≤12.41 Gy	10(83.3 %)	2(16.7 %)	0.013*
	>12.41Gy	4(33.3 %)	8(66.7 %)	
D_10%_	≤8.75Gy	7(58.3 %)	5(41.7 %)	1
	>8.75 Gy	7(58.3 %)	5(41.7 %)	
D_20%_	≤8.22 Gy	7(58.3 %)	5(41.7 %)	1
	>8.22 Gy	7(58.3 %)	5(41.7 %)	
D_30%_	≤7.94 Gy	7(58.3 %)	5(41.7 %)	1
	>7.94 Gy	7(58.3 %)	5(41.7 %)	
D_40%_	≤7.70 Gy	7(58.3 %)	5(41.7 %)	1
	>7.70 Gy	7(58.3 %)	5(41.7 %)	
D_50%_	≤7.48 Gy	7(58.3 %)	5(41.7 %)	1
	>7.48 Gy	7(58.3 %)	5(41.7 %)	
D_80%_	≤6.73 Gy	7(58.3 %)	5(41.7 %)	1
	>6.73 Gy	7(58.3 %)	5(41.7 %)	
D_100%_	≤5.73 Gy	7(58.3 %)	5(41.7 %)	1
	>5.73 Gy	7(58.3 %)	5(41.7 %)	

The status of neurocognitive change shown here is tailored to patients’ performance on Wisconsin Card Sorting Test – Perseverative Errors 4 months after the HS-WBRT course in 24 patients

The N (%) is listed to represent the number of patients and its corresponding percentage

* indicates that statistical significance is noted

To summarize the statistically significant findings in our initial analyses by using chi-square test, it was noted that the EQD_2_ of maximal dose, D_10%_, D_50%,_ D_80%_, and minimal dose delivered to bilateral hippocampi with <12.60 Gy, <8.81 Gy, <7.45 Gy, <6.80 Gy and <5.83 Gy respectively were significantly associated with functional preservation in Wechsler Memory Scale-III Word List (WMS-WL) immediate recall (*p*-values =0.004, 0.041, 0.041, 0.041 and 0.041, respectively) as listed in Table 3. Similarly, the above correlations also existed between hippocampal dosimetry specific to left hippocampus and functional preservation in immediate recall of WMS-WL. By contrast, there were no any significant associations observed between the dosimetric parameters tailored to right hippocampus and verbal functional preservation indicated by WMS-WL, as we had anticipated.

Besides, it was not found that there were any statistically significant dosimetric correlations with neurocognitive preservation of visual reproduction governed generally by right hippocampus (Data not shown). Moreover, as shown in Table 4, the EQD_2_ of maximal dose irradiating left hippocampus with <12.41 Gy was significantly associated with functional preservation in preservative errors of Wisconsin Card Sorting Test (*p*-value =0.013).

**Fig. 4:**
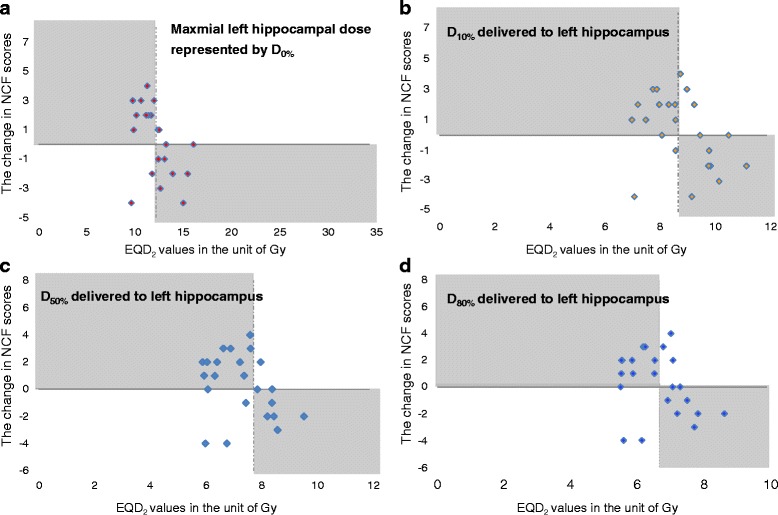
Scatter plots displaying the association between hippocampal dosimetry and neurocognitive functions. Each colored dot represents the change in the NCF scores obtained between before and after the HS-WBRT course for each individual patient. Horizontal axis indicates the hippocampal dosimetry irradiating left hippocampus in a unit of Gy converted to the equivalent dose in 2-Gy fractions (EQD_2_). Vertical axis represents the extent of the NCF change; negative values indicate that there is a specific neurocognitive decline in immediate recall of verbal memory (WLL-IR) after the course of HS-WBRT. Of note, a dotted vertical line in each panel stands for the median dose irradiating left hippocampus in a unit of Gy (EQD_2_). Moreover, the shadowed areas in each panel support the theoretical hypothesis that the higher EQD_2_ the hippocampus receives, the less likely the corresponding NCF is to be spared

As illustrated in Fig 4, it provides us a direct visualization of how hippocampal dosimetry might correlate with neurocognitive change after the course of HS-WBRT with several scatter plots. Theoretically thinking, the higher EQD_2_ the hippocampus receives, the less likely the responsible neurocognitive function is to be preserved. Herein, only the scatter plots concerning the hippocampal dosimetry delivered to left hippocampus are selectively displayed. It is shown that among the dose levels of maximal dose, D_10%_ D_40%_, D_50%_, and D_80%_ delivered to left hippocampus, the lower the corresponding EQD_2_ is, the more likely left hippocampus-dependent verbal memory would be preserved according to the specific test of Word List Learning-Immediate Recall (WLL-IR).

Based on the binary logistic regression analyses performed to examine the potential impacts of hippocampal dosimetric parameters on NCF change after the course of HS-WBRT, it seemed that dosimetric parameters specific to left sided hippocampus exerted an influence on immediate recall of verbal memory rather than its long-term delayed recall and recognition counterparts. As demonstrated in Table 5, the minimal dose irradiating left hippocampus indeed imposes an independent effect on the specific neurocognitive function, immediate recall of verbal memory (WLL-IR). Similarly, the mean dose delivered to left hippocampus also confers a statistically significant influence on immediate recall of verbal function. Quantitatively, as the above mean dose (converted to EQD_2_) increases by one more Gy, there would be a 4-fold increase approximately in the risk for patients in our study to experience a neurocognitive decline in immediate recall of verbal memory (WLL-IR) after the HS-WBRT course (adjusted odds ratio, 4.08; *p*-value, 0.042; 95 % confidence interval, 1.055 – 15.780). Nevertheless, as for the dosimetric parameters tailored to right side hippocampus, it seemed that there were no observed independent effects of right hippocampus-specific dosimetric parameters on verbal or non-verbal memory (Data not presented).

**Table 5:** Binary regression analyses addressing the impact of hippocampal dosimetric parameters on patients’ verbal memory

NCF tests	Dosimetric parameters	Crude OR (*p*-value)	Adjusted OR	Statistical significance	95 % CI
WLL-IR					
	R-hippo mean	2.232 (*p* = 0.108)	2.901	0.077	0.893–9.424
	R-hippo max	1.026 (*p* = 0.856)	1.040	0.802	0.763–1.419
	R-hippo min	4.380 (*p* = 0.077)	5.818	0.061	0.920–36.805
	L-hippo mean	2.870 (*p* = 0.060)	4.080	0.0420*	1.055–15.780
	L-hippo max	1.003 (*p* = 0.975)	1.012	0.920	0.802–1.276
	L-hippo min	7.903 (*p* = 0.037*)	14.345	0.040*	1.126–182.689
	B-hippo mean	2.569 (*p* = 0.076)	3.510	0.052	0.989–12.460
	B-hippo max	1.004 (*p* = 0.974)	1.011	0.930	0.797–1.282
	B-hippo min	5.333 (*p* = 0.070)	8.147	0.060	0.917–72.401
WLL-LTDR					
	R-hippo mean	1.114 (*p* = 0.805)	1.169	0.769	0.413–3.313
	R-hippo max	0.894 (*p* = 0.551)	0.883	0.652	0.513–1.519
	R-hippo min	1.203 (*p* = 0.784)	1.339	0.716	0.277–6.481
	L-hippo mean	1.218 (*p* = 0.665)	1.366	0.581	0.452–4.129
	L-hippo max	0.926 (*p* = 0.596)	0.973	0.864	0.711–1.332
	L-hippo min	1.784 (*p* = 0.352)	2.249	0.322	0.453–11.178
	B-hippo mean	1.176 (*p* = 0.718)	1.291	0.643	0.438–3.809
	B-hippo max	0.918 (*p* = 0.574)	0.938	0.738	0.644–1.366
	B-hippo min	1.349 (*p* = 0.672)	1.612	0.584	0.292–8.897
WLL-R					
	R-hippo mean	0.994 (*p* = 0.990)	1.262	0.699	0.389–4.093
	R-hippo max	1.321 (*p* = 0.183)	3.091	0.147	0.671–14.232
	R-hippo min	1.481 (*p* = 0.616)	1.966	0.430	0.367–10.528
	L-hippo mean	1.177 (*p* = 0.757)	1.573	0.460	0.473–5.234
	L-hippo max	1.370 (*p* = 0.209)	2.290	0.084	0.894–5.868
	L-hippo min	1.669 (*p* = 0.465)	2.199	0.357	0.412–11.746
	B-hippo mean	1.089 (*p* = 0.870)	1.424	0.563	0.430–4.718
	B-hippo max	1.309 (*p* = 0.214)	2.308	0.114	0.817–6.521
	B-hippo min	1.632 (*p* = 0.551)	2.273	0.363	0.388–13.305

Abbreviations: *WLL* word list learning of Wechsler memory scale-III, *IR* immediate recall, *LTDR* long term delayed recall, *R* recognition, *R-hippo* right hippocampus, *L-hippo* left hippocampus, *B-hippo* bilateral hippocampi as a composite structure, *OR* odds ratio, *CI* confidence Interval

The NCF test results shown here are derived from of Wechsler Memory Scale-III Word Lists

Adjusted OR represents the independent effect of the dosimetric parameter of interest after controlling for the patient’s age at study recruitment and whether craniotomy with tumor removal was performed or not before the referral to our Department of Radiation Oncology

The status of neurocognitive change shown here is according to patients’ performance on verbal memory indicated by Wechsler Memory Scale-III Word Lists

* indicates that statistical significance is noted

## Discussion

### Significance of this prospective study

Since only one intracranial failure in the current study might be associated with the dose-reducing region indicating the zone of hippocampal avoidance (unpublished results), we believe that reducing the dose irradiating the hippocampal areas when treating patients with WBRT might not compromise intracranial tumor control. Furthermore, several related studies have consistently supported selectively reducing the dose delivered to the hippocampus when treating oligometastatic patients with WBRT [26, 27, 33, 34].

Although our results have clearly shown that specific NCFs (i.e., verbal and non-verbal learning memory, executive functions, and psychomotor speed) do not significantly change or decline in patients who have undergone hippocampal sparing during the WBRT course, our findings might just be applied to the time span of 4 months after HS-WBRT and a decline after this time point cannot be utterly excluded. Nevertheless, the changes in most of the specific NCFs assessed in our study tend to stabilize and even improve rather decline as what have usually been anticipated.

### Meaningful implications arising from hippocampal dosimetry analyses

In the study reported by Gondi et al. [16], it was documented that the biologically equivalent dose in 2-Gy fractions (EQD_2_) to 40 % of the composite structure of bilateral hippocampi greater than 7.3 Gy was significantly associated with neurocognitive impairment regarding the delayed recall of WMS-WL. Looking back to our current study which also adopted the same verbal learning test (WMS-WL), it is indeed observed that the dosimetric parameters concerning particularly left hippocampus imposes an independent influence on the immediate recall of verbal memory rather than its delayed recall or recognition counterparts. Nevertheless, our results do support the hypothesis that verbal memory is likely to be impaired by local irradiation in a dose–response relationship.

By contrast, we failed to find any correlations existing between hippocampal dosimetry and the non-verbal memory function indicated by the NCF test of visual reproduction (WMS-VR). Such negative findings are quite consistent with those reported by Gondi et al. [16]; and therefore some plausibility might be proposed to potentially explain the above negative results. First, non-verbal memory learning is less likely to be hampered after exposure to irradiation or more likely to be compensated for by other related neurocognitive functions and circuits. Second, it is possible that the administered NCF test (WMS-VR) is too insensitive to detect the subtle non-verbal memory decline after the course of HS-WBRT, during which only a median EQD_2_ of 7.68 Gy converted to a biologically equivalent dose in 2-Gy fractions is delivered to 40 % of the composite hippocampal structure. Indeed, the past evidence has proved that the spatial learning ability is significantly associated with the hippocampal functions [35]. In addition, participants’ performance on WMS-VR mainly requires specific recalls from the given geometric shapes, which do not necessarily involve the information of spatial relations. Therefore, the negative findings concerning the impact of dosimetric parameters on the scores of WMS-VR might be reasonable.

### Strengths and potential limitations of the present study

To the best of our knowledge, there has been little evidence investigating neurocognitive outcomes for non-primary brain tumor patients, let alone cancer patients with brain metastasis in Taiwanese population. Therefore, this prospective clinical study with neurocognitive outcome research might be a pioneering study focusing on neurocognitive outcomes in the field of neuro-oncology in an eastern Asian country. Moreover, all contouring tailored to the imaging anatomy of the hippocampus was consistently delineated and verified by the same neuroimaging radiologist instead of neurosurgeons or radiation oncologists only. Similarly, all neurocognitive assessments were administered under the supervision of an experienced neuropsychologist, who had selected a neurobehavioral test battery specifically for the current study. By virtue of this prospective study carried out in Taiwan, we can not only acquire both neurocognitive and neuro-oncological results, but also have the great opportunity to correlate hippocampal dose-volume histogram (DVH) data with neurocognitive functional outcomes. Definitely these objective results will guide us helpfully when formulating and designing the future study protocols.

Even though the current study has apparently shown that neurocognitive functions tended to stabilize instead of decline after HS-WBRT, we should always be aware that patients experiencing perhaps the worst neurocognitive status due to any reasons after HS-WBRT might fail to receive the neurocognitive follow-up assessment because of non-compliance. As a consequence, we have to interpret our neurocognitive data more cautiously in order not to be masked by such a potential bias. Besides, even though this prospective study might be limited by the fact that there is no real control group for which conventional WBRT without hippocampal sparing is delivered, actually each patient indeed serves as his/her own control, because the difference in scores obtained at baseline and at pre-specified post-treatment intervals will be measured and calculated. The patient population in this prospective study actually reflects the real world patient management in our country, where WBRT combined with/without surgical removal instead of upfront radiosurgery can generally be reimbursed by national insurance when managing cancer patients with newly-diagnosed oligometastatic brain disease or at a higher risk of brain metastasis [36]. Therefore, we enroll two main patient subgroups; one subgroup stands for patients referred for PCI and the other represents those with a limited burden of brain metastases (unpublished results).

### Neurocognitive status/change before and after HS-WBRT

Multi-domains of NCF were examined in this study, and our results consequently showed no significant differences or declines in verbal or non-verbal memory, executive functions and psychomotor speed between baseline and 4-months after the start of HS-WBRT course; it is also found that the change in multi-domains of NCF generally tend to become stabilized with somewhat improvement. As a matter of fact, our findings support that the conformal sparing of hippocampal areas during the delivery of WBRT might preserve patients’ NCF mostly or alleviate the extent of NCF changes at least [26, 37]. Indeed, it has been documented that WBRT is associated with late neurotoxicity resulting from brain irradiation and will induce multi-faceted difficulties in patients including memory, attention and motor control [8, 38]; therefore, HS-WBRT was developed to preserve cortical NCFS in patients receiving cranial RT [20, 26, 39]. Although quite a few researches [33, 40, 41] have conceptually hypothesized that HS-WBRT might mitigate the cognitive decline after brain RT, studies concerning the dynamic changes of NCFs before and after HS-WBRT are still limited and preliminary. For example, Gondi et al. have conducted a phase II clinical trial, RTOG 0933, to investigate the effects of HS-WBRT on patients with brain metastases and they evaluated memory performances by using the Hopkins Verbal Learning Test (HVLT) at 4 months after initiating brain RT [22]. Our prospective study further provides convincing and promising evidence that specific NCFs in patients with oligometastatic brain disease are quite stable between the baseline assessment and those evaluated at 4 months after HS-WBRT.

### Future directions

First of all, the dosimetric parameters utilized in the current study are mainly biologically equivalent doses in 2-Gy fractions (EQD_2_) assuming an assuming an α/β value of 2 Gy. In fact, the concept of equivalent uniform dose (EUD) has been introduced in recent years [42, 43] in order to determine the radiation exposure of the hippocampal structure more accurately. In the future, it deserves investigating the correlations of EUD of the hippocampus in patients receiving cranial irradiation with relevant neurocognitive changes after HS-WBRT. In addition, given the ability of modern VMAT techniques to spare the hippocampal structures in excess of the dosimetric threshold during the 30-Gy WBRT course, we are going to explore the dosimetric findings in ongoing and future prospective studies of hippocampal sparing during cranial irradiation.

Second, whether the current study can be expended to a larger-scale prospective study with introducing an appropriate control group deserves detailed discussion and debates. Conceptually, patients fitting the same eligibility and randomized to receive conventional WBRT without hippocampal sparing should be the ideal control patient population. However, ethical considerations cannot be avoided at all provided that HS-WBRT could achieve similar oncological outcomes, more favorable neurocognitive outcomes without violating the safety profile, as compared with conventional WBRT [22].

## Conclusions

Neurocognitive assessments provide further help for neuro-oncologists and relevant health professionals when managing cancer patients harboring brain metastases. Additionally, both intracranial control and satisfactory functional preservation by reducing the dose irradiating the hippocampus during the WBRT course have been achieved in the current study. The correlation between hippocampal dosimetry and neurocognitive outcomes would definitely guide us when formulating and designing the future study protocols. Providing that modern VMAT techniques can reduce the dose irradiating the hippocampus below dosimetric threshold, patients should be recruited in prospective trials of hippocampal sparing during cranial irradiation in order to accomplish neurocognitive preservation while maintaining intracranial disease control.

### Ethics approval

The study protocol had been approved by the institutional review board (IRB) at Chang Gung Medical Foundation (IRB 101-4151B and 103-1090C).

### Consent for publication

Not applicable because this manuscript does not contain any data about individual persons.

## Additional file

Additional file 1:
**A summary of patient demographics, tumor and disease characteristics.** (PDF 86 kb)

## Abbreviations

NCF: Neurocognitive Function
SCLC: small cell lung cancer
VMAT: Volumetric Modulated Arc Therapy
WBRT: Whole Brain Radiotherapy
HS-WBRT: Hippocampus Sparing during Whole Brain Radiotherapy
HA: Hippocampal avoidance
DVH: Dose Volume Histogram
RPA: Recursive partitioning analysis
PCI: prophylactic cranial irradiation
EQD_2_: Equivalent Biologically equivalent doses in 2-Gy fractions
WLL: Word List Learning
VR: Visual Reproduction
MCST: Modified Card Sorting Test
WMS: Wechsler Memory Scale
WAIS: Wechsler Adult Intelligence Test
PSI: Psychomotor Speed Index

## References

[1] Norden AD, Wen PY, Kesari S (2005). Brain metastases. Curr Opin Neurol.

[2] Koay E, Sulman EP (2012). Management of brain metastasis: past lessons, modern management, and future considerations. Curr Oncol Rep.

[3] Maclean J, Fersht N, Singhera M, Mulholland P, McKee O, Kitchen N (2013). Multi-disciplinary management for patients with oligometastases to the brain: results of a 5 year cohort study. Radiat Oncol.

[4] Slotman B, Faivre-Finn C, Kramer G, Rankin E, Snee M, Hatton M (2007). Prophylactic cranial irradiation in extensive small-cell lung cancer. N Engl J Med.

[5] Aoyama H, Tago M, Kato N, Toyoda T, Kenjyo M, Hirota S (2007). Neurocognitive function of patients with brain metastasis who received either whole brain radiotherapy plus stereotactic radiosurgery or radiosurgery alone. Int J Radiat Oncol Biol Phys.

[6] Kocher M, Soffietti R, Abacioglu U, Villa S, Fauchon F, Baumert BG (2011). Adjuvant whole-brain radiotherapy versus observation after radiosurgery or surgical resection of one to three cerebral metastases: results of the EORTC 22952–26001 study. J Clin Oncol.

[7] Sun A, Bae K, Gore EM, Movsas B, Wong SJ, Meyers CA (2011). Phase III trial of prophylactic cranial irradiation compared with observation in patients with locally advanced non-small-cell lung cancer: neurocognitive and quality-of-life analysis. J Clin Oncol.

[8] Welzel G, Fleckenstein K, Schaefer J, Hermann B, Kraus-Tiefenbacher U, Mai SK (2008). Memory function before and after whole brain radiotherapy in patients with and without brain metastases. Int J Radiat Oncol Biol Phys.

[9] Scoville WB, Milner B (2000). Loss of recent memory after bilateral hippocampal lesions. 1957. J Neuropsychiatry Clin Neurosci.

[10] Hellstrom NA, Bjork-Eriksson T, Blomgren K, Kuhn HG (2009). Differential recovery of neural stem cells in the subventricular zone and dentate gyrus after ionizing radiation. Stem Cells.

[11] Mizumatsu S, Monje ML, Morhardt DR, Rola R, Palmer TD, Fike JR (2003). Extreme sensitivity of adult neurogenesis to low doses of X-irradiation. Cancer Res.

[12] Raber J, Rola R, LeFevour A, Morhardt D, Curley J, Mizumatsu S (2004). Radiation-induced cognitive impairments are associated with changes in indicators of hippocampal neurogenesis. Radiat Res.

[13] Rola R, Raber J, Rizk A, Otsuka S, VandenBerg SR, Morhardt DR (2004). Radiation-induced impairment of hippocampal neurogenesis is associated with cognitive deficits in young mice. Exp Neurol.

[14] Lam LC, Leung SF, Chan YL (2003). Progress of memory function after radiation therapy in patients with nasopharyngeal carcinoma. J Neuropsychiatry Clin Neurosci.

[15] Ali AN, Ogunleye T, Hardy CW, Shu HK, Curran WJ, Crocker IR (2014). Improved hippocampal dose with reduced margin radiotherapy for glioblastoma multiforme. Radiat Oncol.

[16] Gondi V, Hermann BP, Mehta MP, Tome WA (2012). Hippocampal dosimetry predicts neurocognitive function impairment after fractionated stereotactic radiotherapy for benign or low-grade adult brain tumors. Int J Radiat Oncol Biol Phys.

[17] Marsh JC, Ziel GE, Diaz AZ, Wendt JA, Gobole R, Turian JV (2013). Integral dose delivered to normal brain with conventional intensity-modulated radiotherapy (IMRT) and helical tomotherapy IMRT during partial brain radiotherapy for high-grade gliomas with and without selective sparing of the hippocampus, limbic circuit and neural stem cell compartment. J Med Imaging Radiat Oncol.

[18] Han G, Liu D, Gan H, Denniston KA, Li S, Tan W (2014). Evaluation of the dosimetric feasibility of hippocampal sparing intensity-modulated radiotherapy in patients with locally advanced nasopharyngeal carcinoma. PLoS One.

[19] Khodayari B, Michaud AL, Stanic S, Wooten OH, Dublin A, Purdy JA (2014). Evaluation of hippocampus dose for patients undergoing intensity-modulated radiotherapy for nasopharyngeal carcinoma. Br J Radiol.

[20] Ghia A, Tome WA, Thomas S, Cannon G, Khuntia D, Kuo JS (2007). Distribution of brain metastases in relation to the hippocampus: implications for neurocognitive functional preservation. Int J Radiat Oncol Biol Phys.

[21] Truc G, Martin E, Mirjolet C, Chamois J, Petitfils A, Crehange G (2013). The role of whole brain radiotherapy with hippocampal-sparing. Cancer Radiother.

[22] Gondi V, Pugh SL, Tome WA, Caine C, Corn B, Kanner A (2014). Preservation of memory with conformal avoidance of the hippocampal neural stem-cell compartment during whole-brain radiotherapy for brain metastases (RTOG 0933): a phase II multi-institutional trial. J Clin Oncol.

[23] Gondi V, Tolakanahalli R, Mehta MP, Tewatia D, Rowley H, Kuo JS (2010). Hippocampal-sparing whole-brain radiotherapy: a “how-to” technique using helical tomotherapy and linear accelerator-based intensity-modulated radiotherapy. Int J Radiat Oncol Biol Phys.

[24] Oskan F, Ganswindt U, Schwarz SB, Manapov F, Belka C, Niyazi M (2014). Hippocampus sparing in whole-brain radiotherapy. A review. Strahlentherapie Und Onkologie.

[25] Prokic V, Wiedenmann N, Fels F, Schmucker M, Nieder C, Grosu AL (2013). Whole brain irradiation with hippocampal sparing and dose escalation on multiple brain metastases: a planning study on treatment concepts. Int J Radiat Oncol Biol Phys.

[26] Marsh JC, Herskovic AM, Gielda BT, Hughes FF, Hoeppner T, Turian J (2010). Intracranial metastatic disease spares the limbic circuit: a review of 697 metastatic lesions in 107 patients. Int J Radiat Oncol Biol Phys.

[27] Wan JF, Zhang SJ, Wang L, Zhao KL (2013). Implications for preserving neural stem cells in whole brain radiotherapy and prophylactic cranial irradiation: a review of 2270 metastases in 488 patients. J Radiat Res.

[28] Gaspar L, Scott C, Rotman M, Asbell S, Phillips T, Wasserman T (1997). Recursive partitioning analysis (RPA) of prognostic factors in three Radiation Therapy Oncology Group (RTOG) brain metastases trials. Int J Radiat Oncol Biol Phys.

[29] Gaspar LE, Scott C, Murray K, Curran W (2000). Validation of the RTOG recursive partitioning analysis (RPA) classification for brain metastases. Int J Radiat Oncol Biol Phys.

[30] Castilla-Ortega E, Pedraza C, Estivill-Torrus G, Santin LJ (2011). When is adult hippocampal neurogenesis necessary for learning? evidence from animal research. Rev Neurosci.

[31] Nelson HE (1976). A modified card sorting test sensitive to frontal lobe defects. Cortex.

[32] Wechsler D (1997). Wechsler Adult Intellegence Scale - Third Edition.

[33] Gondi V, Tome WA, Marsh J, Struck A, Ghia A, Turian JV (2010). Estimated risk of perihippocampal disease progression after hippocampal avoidance during whole-brain radiotherapy: safety profile for RTOG 0933. Radiother Oncol.

[34] Harth S, Abo-Madyan Y, Zheng L, Siebenlist K, Herskind C, Wenz F (2013). Estimation of intracranial failure risk following hippocampal-sparing whole brain radiotherapy. Radiother Oncol.

[35] Eichenbaum H (2000). A cortical-hippocampal system for declarative memory. Nat Rev Neurosci.

[36] Tang SG, Tseng CK, Tsay PK, Chen CH, Chang JW, Pai PC (2005). Predictors for patterns of brain relapse and overall survival in patients with non-small cell lung cancer. J Neurooncol.

[37] Barani IJ, Cuttino LW, Benedict SH, Todor D, Bump EA, Wu Y (2007). Neural stem cell-preserving external-beam radiotherapy of central nervous system malignancies. Int J Radiat Oncol Biol Phys.

[38] Roman DD, Sperduto PW (1995). Neuropsychological effects of cranial radiation: current knowledge and future directions. Int J Radiat Oncol Biol Phys.

[39] Gutierrez AN, Westerly DC, Tome WA, Jaradat HA, Mackie TR, Bentzen SM (2007). Whole brain radiotherapy with hippocampal avoidance and simultaneously integrated brain metastases boost: a planning study. Int J Radiat Oncol Biol Phys.

[40] Barani IJ, Benedict SH, Lin PS (2007). Neural stem cells: implications for the conventional radiotherapy of central nervous system malignancies. Int J Radiat Oncol Biol Phys.

[41] Barani IJ, Larson DA, Berger MS (2013). Future directions in treatment of brain metastases. Surg Neurol Int.

[42] Bodensohn R, Soehn M, Ganswindt U, Schupp G, Nachbichler SB, Schnell O (2014). Hippocampal EUD in primarily irradiated glioblastoma patients. Radiat Oncol.

[43] Niemierko A (1997). Reporting and analyzing dose distributions: A concept of equivalent uniform dose. Med Phys.

