# Supplementary material for: Hippocampal dosimetry correlates with the change in neurocognitive function after hippocampal sparing during whole brain radiotherapy: a prospective study
Source: Radiat Oncol. 2015 Dec 10;10:253. doi: 10.1186/s13014-015-0562-x (PMC4676088; doi:10.1186/s13014-015-0562-x)
Supplement: Additional file 1: — A summary of patient demographics, tumor and disease characteristics. (PDF 86 kb) [file 13014_2015_562_MOESM1_ESM.pdf]

Table 1. Summary of patient demographics, tumor and disease characteristics

Characteristics	No. of patients (%)	No. of lesions (%)
**Number of patients**	40	
**Number of brain metastatic lesions**		49
**Age at registration**, years		
Median	57.3	
Mean (range)	58.3 (28.3 – 79.1)	
**Gender**		
Male	20 (50%)	
Female	20 (50%)	
**Performance status before the course of brain RT**		
KPS ≥90	15 (37.5%)	
70 ≤KPS <90	25 (62.5%)	
**Performance status (ECOG)**		
0-1	31 (77.5%)	
2	9 (22.5%)	
**Histological subtype of primary cancer**		
Lung, NSCLC	17 (42.5%)	
Lung, SCLC	5 (12.5%)	
Breast	9 (22.5%)	
Others[a]	9 (22.5%)	
**Number of brain metastatic lesions at diagnosis**		
0, PCI	3 (7.5%)	
1	28 (70%)	
2	6 (15 %)	
3	3 (7.5%)	
**Number of surgical cavities**		31
**Extent of resection**		
En-bloc gross total		28 (90.3%)
Subtotal		3 (9.7%)
**Role of WBRT**		
PCI	3 (7.5%)	
Adjuvant post craniotomy	23 (57.5%)	31(63.3%)
Therapeutic for oligometastatic	14(35%)	18(36.7%)

brain disease[b]	
**Status of extracranial metastasis**	
Stable/Controlled	24 (60%)
Uncontrolled/Progressive	16 (40%)
**RTOG RPA class**[c]	
Class I	12 (32.4%)
Class II	25 (67.6%)

[a]One case with retroperitoneal germ cell tumor (embryonal carcinoma), two cases with hepatocellular carcinoma (HCC), one case with esophageal cancer, one case with neuroendocrine carcinoma of uterine cervix, one case with malignant melanoma, and one case with liver angiosarcoma, one case with endometrial adenocarcinoma, one case with bladder cancer, and one case with renal cell carcinoma.

[b]According to our predefined criteria, oligometastatic brain disease indicates that the number of brain metastatic lesions is three or less shown on brain MRI.

[c]Based on recursive partitioning analyses (RPA) performed by Radiation Therapy Oncology Group (RTOG) tailored to cancer patients with brain metastasis, three classes were suggested: Class I: patients with KPS greater than or equal to 70, < 65 years of age with controlled primary and no extracranial metastases; Class III: KPS < 70; Class II: all others.
